# Improper High‐*T*_c_ Perovskite Ferroelectric with Dielectric Bistability Enables Broadband Ultraviolet‐to‐Infrared Photopyroelectric Effects

**DOI:** 10.1002/advs.202301064

**Published:** 2023-04-23

**Authors:** Lina Hua, Jiaqi Wang, Yi Liu, Wuqian Guo, Yu Ma, Haojie Xu, Shiguo Han, Junhua Luo, Zhihua Sun

**Affiliations:** ^1^ State Key Laboratory of Structure Chemistry Fujian Institute of Research on the Structure of Matter Chinese Academy of Sciences Fuzhou Fujian 350002 P. R. China; ^2^ University of Chinese Academy of Sciences Beijing 100049 P. R. China; ^3^ Fujian Science and Technology Innovation Laboratory for Optoelectronic Information of China Fuzhou Fujian 350108 P. R. China

**Keywords:** broadband photopyroelectric effects, dielectric bistability, hybrid perovskites, improper ferroelectrics

## Abstract

The photopyroelectric effect in ferroelectrics has shown great potential for application in infrared detection and imaging. One particular subclass is broadband with dielectric bistability, which allows for large pyroelectric figures‐of‐merit (FOMs). Herein, an improper high‐*T*_c_ perovskite ferroelectric, (IA)_2_(EA)_2_Pb_3_Cl_10_ (**1**, where IA is isoamylammonium and EA is ethylammonium) is presented, in which spontaneous polarization (*P*_s_) stems from the dynamic ordering of organic cations and the tilting of distorted PbCl_6_ octahedra. Notably, **1** displays unusual dielectric bistability with small variations in the temperature‐dependent dielectric constants near *T*_c_ = 392 K; this bistable attribute endows large pyroelectric FOMs with peak voltage efficiency (*F*_V_ = 1.7×10^−2^ cm^2^ µC^−1^) and sensitivity (*F*_D_ = 3.9×10^−4^ Pa^−1/2^). These *F*_V_ and *F*_D_ parameters, beyond those of their proper counterparts, make **1** a promising candidate for infrared photodetection. As expected, the broadband photopyroelectric effects observed in **1** covered the ultraviolet to infrared‐II spectral region (266–1950 nm). Such *P*_s_‐directed photoactivities overcome the optical bandgap limitation and allow for wide‐wave photodetection. As an innovative study on improper ferroelectricity, light is shaded here on the targeted engineering of new electrically ordered candidate materials for smart optoelectronic devices.

## Graphical abstract

It is demonstrated that improper ferroelectrics enable broadband photopyroelectric effects covering an ultraviolet to infrared‐II spectral region (266−1950 nm), which breaks the limitation of the optical bandgap. Light is shaded here on the targeted engineering of new electric‐ordered candidates for smart wide‐wave photodetection devices.

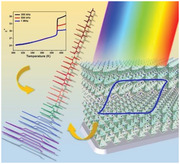

## 1 Introduction

Ferroelectric materials are a subclass of pyroelectric substances that have many technological device applications, such as energy irradiation detection, thermal imaging, and infrared detection.^[^ ^1^, ^2^ ^]^ The basic principle is that, under a small temperature variation, the intrinsic pyroelectric effect of ferroelectric materials converts thermal energy to electrical signals and directly creates visible current. This thermal‐to‐electrical process has the merits of high sensitivity, low‐cost energy consumption, and a fast response.^[^ ^3^, ^4^ ^]^ Conceptually, the realization of efficient conversion requires large pyroelectric figures‐of‐merit (FOMs) that depend on several independent physical parameters, including the pyroelectric coefficient (*p*_e_), specific heat capacity (*c*_v_), dielectric constant, and dielectric loss.^[^ ^5^ ^]^ As exemplified by the small‐area (point) pyroelectric detector, two crucial FOMs are voltage responsivity *F*_V_ = *p_e_*/(*ε*_r_ *ε*_0_ *c*_v_) and detection sensitivity *F*_D_ = *p_e_*/(*c*_v_(*ε*_r_ *ε*_0_tan*δ*)^1/2^, where *ε*_r_ and tan*δ* are relative dielectric constant and dielectric loss, respectively. *F*_v_ is an indicator used to evaluate the voltage output of pyroelectric materials used for infrared detection.^[^ ^3^, ^6^ ^]^ Because the detection limit is related to dielectric loss, *F*_D_ characterizes the capability of perceiving weak thermal signals that compete with noise.^[^ ^7^ ^]^ These two expressions suggest that high‐performance devices require pyroelectric materials with large *p*_e_, low *ε*_r_ and tan*δ*, and small specific heat values, over a broad temperature range.

During phase transitions in proper ferroelectrics, where spontaneous polarization (*P*_s_) is the primary order parameter, *P*_s_ in improper ferroelectrics is no longer a spontaneously generated order parameter but a secondary parameter induced by structural distortion, spin ordering, or charge ordering.^[^ ^8^, ^9^, ^10^ ^]^ Generally, the dielectric constants of improper ferroelectrics only exhibit a minor increase near *T*_c_, which is usually at least one order of magnitude lower than that of proper ferroelectrics.^[^ ^10^, ^11^ ^]^ Dielectric bistability refers to multistable and switchable dielectric states that exhibit almost temperature‐independent behavior during phase transitions. Owing to the relatively small dielectric constants, this might be a potential hint for acquiring improper ferroelectricity, which allows for dramatic pyroelectric FOMs. For typical oxide ferroelectrics (e.g., BaTiO_3_, PbZrO_3_, and PbTiO_3_) ,^[^ ^12^, ^13^, ^14^, ^15^ ^]^ the large dielectric constants are inferior to those of pyroelectric FOMs for practical applications, particularly near the phase transition critical zone. Alternatively, molecular ferroelectrics with low *ε*_r_ values are emerging as supplements for their inorganic counterparts, such as triglycine sulfate (TGS), deuterated TGS (DTGS), and polyvinylidene fluoride (PVDF).^[^ ^7^, ^16^ ^]^ Intensive efforts have been made to promote the pyroelectric FOMs of molecular compounds using chemical doping or modification.^[^ ^17^, ^18^, ^19^ ^]^ Emphatically, the dielectric bistability of improper ferroelectrics is envisaged as a competitive strategy in this paradigm.^[^ ^20^ ^]^ The combination of ferroelectric *P*_s_, low permittivity, and small variations in dielectric constants, facilitates high pyroelectric FOMs, thereby behaving as ideal candidates for thermal detection.^[^ ^10^ ^]^ Moreover, a broad operating temperature range is crucial for pyroelectric detection, which requires high‐*T*_c_ ferroelectric candidates.^[^ ^21^ ^]^ Hence, designing new high‐*T*_c_ improper ferroelectric materials with bistable dielectric activities is important.

A recently emerging family of improper ferroelectrics is molecular perovskites ,^[^ ^22^ ^]^ as validated by metal‐halide perovskites ,^[^ ^11^, ^23^ ^]^ metal formates ,^[^ ^24^, ^25^ ^]^ and metal‐free molecules ,^[^ ^6^, ^26^ ^]^ which have the infinite structural flexibility allowed by organic ingredients. The combination of multipolar ordering (organic moieties) and octahedral distortion (rigid inorganic blocks) is an established method for acquiring improper ferroelectricity. Inspired by this principle, we have acquired a 2D perovskite improper ferroelectric, (IA)_2_(EA)_2_Pb_3_Cl_10_ (**1,** where IA is isoamylammonium and EA is ethylammonium), which shows a high‐*T*_c_ symmetry breaking at *T*_c_ = 392 K along with ferroelectric *P*_s_ of ≈1.2 µC cm^−2^, which is attributed to the multipolar ordering of organic cations and tilting of distorted PbCl_6_ octahedra. In particular, **1** exhibited an extremely small variation in the dielectric constants over a wide temperature range, revealing its improper ferroelectricity. This leads to its exceptional pyroelectric behavior, including high peak FOMs of voltage efficiency (*F*_V_ = 1.7×10^−2^ cm^2^ µC^−1^) and sensitivity (*F*_D_ = 3.9×10^−4^ Pa^−1/2^) near *T*_c_. Consequently, the optical bandgap limitation is overcome by the broadband photopyroelectric effects that are reasonably explained in **1** and cover the ultraviolet (UV) to the infrared‐II (NIR‐II) spectral region (266–1950 nm). These results make **1** a promising candidate for high‐performance infrared photodetectors and demonstrate that dielectric bistability is a useful method for using novel electrically ordered candidates.

## 2 Results and Discussion

### 2.1 Basic Structure Assessment

**Figure 1:**
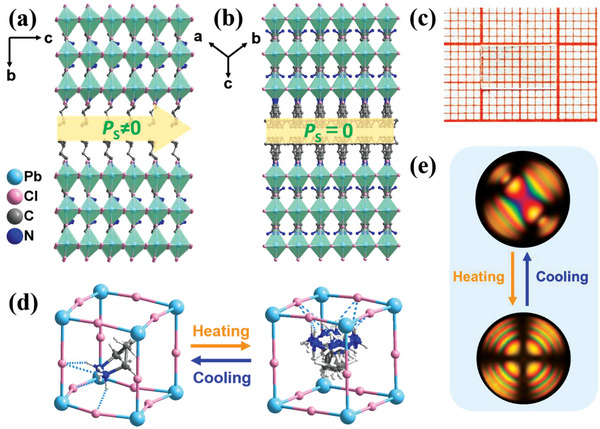
Crystal structures of **1** were collected at different phases. a) Packing diagram as viewed in the direction of the *a*‐axis at 300 K (FEP). The yellow arrowhead indicates the possible *P*_s_ along its *c*‐axis. b) Packing structure observed at 400 K (paraelectric phase, PEP). c) Transparent plate‐like single crystal. d) Comparison of building blocks for inorganic frameworks at FEP (left) and PEP (right), respectively. e) Temperature‐dependence of optical axes during phase transition.

Structural polarity is an essential requirement for ferroelectrics, which require a broken symmetry of the inversion centers. For molecular perovskite analogs, structural flexibility enables different types of freedom in molecular motion, such as (un)conventional tilting ,^[^ ^27^, ^28^ ^]^ columnar shift ,^[^ ^29^ ^]^ and multipolar ordering.^[^ ^3^, ^30^ ^]^ The synergistic action of the two dynamic ingredients provides access to polarity and ferroelectricity, as exemplified by the 2D Ruddlesden‐Popper (RP) phase. For **1**, its perovskite‐type PbCl_6_ octahedra exhibited conventional tilting, whereas the organic IA and EA cations were collectively oriented along the *c*‐axis direction. According to the engineering recipe for improper ferroelectrics, rod‐like and disc‐like ingredients with charges localized at a single atom possess high‐order multipoles, for example, a quadrupole moment.^[^ ^31^ ^]^ Hence, the mixed alloying of multipolar organic cations facilitated the emergence of *P*_s_ in **1**. A temperature‐reduction technique was used to produce high‐quality crystals of **1** from its hydrochloric solution (**Figure** **1**c and Figure S1, Supporting Information), which exhibited high thermal stability up to 460 K (Figure S2, Supporting Information). Single‐crystal structure analyses revealed that **1** crystallizes in the polar space group *Fmm*2 at room temperature (Table S1, Supporting Information) and features a typical 2D RP trilayered perovskite configuration. Figure 1a depicts that its basic motif consists of {EA_2_Pb_3_Cl_10_}_∞_ trilayered sheets and spacing bilayers of organic IA^+^ cations, linked together through strong N—H···Cl hydrogen bonds (Figure S3 and Table S4, Supporting Information). In the cages created by PbCl_6_ octahedra that share their corners, organic EA^+^ cations act as “perovskitizers.” Notably, the PbCl_6_ octahedra display a small‐angle steric distortion compared to the ideal octahedron, as verified by the Cl—Pb—Cl bond angles (Tables S2 and S3, Supporting Information). Moreover, the distorted Pb—Cl—Pb bond angle (≈156°) also caused its polar configuration with different diagonals (Figure 1c). These distorted octahedra are bridged with adjacent blocks to form inorganic perovskite trilayers resembling other homologues, such as (IA)_2_(EA)_2_Pb_3_Br_10_ and EA_4_Pb_3_Cl_10_.^[^ ^11^, ^32^ ^]^ This octahedral distortion suggests the conventional tilting of the rigid perovskite framework. Combined with the dynamic ordering of organic moieties, the positively and negatively charged centers deviate from their equilibrium positions, thus contributing to the generation of molecular dipole moments and bulk electric polarization (as shown by the yellow arrowhead in Figure 1a).

Variable‐temperature structural analyses revealed that **1** belongs to the tetragonal system with a centrosymmetric space group of *I4*/*mmm* in the paraelectric phase (PEP; Table S1, Supporting Information). As shown in Figure 1b, an obvious alteration is that all of the C and N atoms are dispersed along the fourfold rotation axis, and organic cations become highly disordered. The trilayered inorganic frameworks adopt a highly symmetric configuration, as verified by their equivalent Pb—Cl—Pb bond angles. Hence, the symmetric alignment of all the components in **1** satisfies the requirement for crystallographic symmetry above *T*_c_. This variation suggests that the order disordering of organic cations and tilting distortion of inorganic frameworks lead to a phase transition of **1**. The transformation of the crystallographic lattice cells between high‐ and low‐temperature ferroelectric phase (FEP) is illustrated in Figure S4a in the Supporting Information, namely, ***c*** ^FEP^ ≈ ***a*** ^PEP^ + ***b*** ^PEP^, ***b*** ^FEP^ ≈ ***c*** ^PEP^, ***a*** ^FEP^ ≈ ***b*** ^PEP^ − ***a*** ^PEP^. The change in crystal symmetry coincides with the Aizu rule of 4/*mmm*F*mm*2, which is one of the 88 species of FEP transitions.^[^ ^33^, ^34^ ^]^ This symmetry breaking in **1** gave rise to four equivalent [110] directions in PEP (Figure S4, Supporting Information), coinciding with the polar direction in FEP.

Hence, its four equivalent *P*_s_ directions reveal the characteristics of biaxial ferroelectricity that resemble some analogs^[^ ^35^ ^]^ such as BA_2_CsAgBiBr_7_, (IA)_2_(EA)_2_Pb_3_I_10_, and [Hdabco]ClO_4_ (where dabco is 1,4‐diazabicyclo[2.2.2]octane).^[^ ^36^, ^37^, ^38^ ^]^ Besides, this symmetry breaking is accompanied by a change in the optical axes. Figure 1e shows the two optical‐axis directions observed for **1** at 298 K (top). When the temperature rises above *T*_c_, it becomes optically uniaxial with only one optical axis (bottom), which agrees with its symmetry breaking from high‐temperature PEP to low‐temperature FEP during the phase transition.

### 2.2 Phase Transition Behaviors

**Figure 2:**
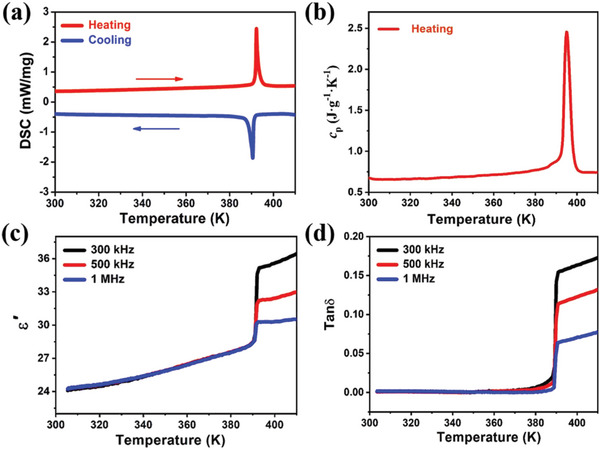
Phase transition properties of **1**. a) DSC curves measured upon heating and cooling. b) Specific heat (*C*_p_) curve in the heating mode. c) Temperature‐dependent dielectric constant (*ε*ʹ) and d) dielectric loss (tan*δ*) were measured at different frequencies.

Symmetric breaking is an indispensable characteristic of most ferroelectrics.^[^ ^35^ ^]^ Primarily, we performed differential scanning calorimetry (DSC) and variable‐temperature dielectric measurements to confirm the phase transition in **1** along with symmetry breaking. The DSC trace shows two exothermic/endothermic thermal peaks at 390/392 K (*T*_c_, Curie temperature), which reveal the reversible phase transition of **1** (**Figure** **2**a). The sharp thermal peaks with obvious hysteresis are reminiscent of first‐order phase transitions.^[^ ^39^ ^]^ Moreover, the specific heat (*C*_p_) trace remains almost stable with the value of 0.68 J g^−1^ K^−1^ but shows a sharp peak in the vicinity of *T*_c_ (Figure 2b). According to the definitions of *F*_D_ and *F*_V_, the relatively small variations in *C*_p_ values would greatly favor the achievement of high FOMs for pyroelectric device applications.

The dielectric constant is a crucial parameter that directly influences the pyroelectric FOMs. Figure 2c shows the temperature‐dependent dielectric spectra of **1** measured along the *c*‐axis. A FEP‐to‐PEP transition occurred at 392 K in conjunction with step‐like dielectric anomalies. Notably, the *ε*_r_ values of **1** displayed a very small change in magnitude from 24 to 34 during the phase transition, and two stable dielectric plateaus with low energies were obtained upon heating (Figure 2c). Comparing the *ε*_r_ values to those of typical inorganic ferroelectrics, such as BaTiO_3_ (*ε*_r_ 5000) and lead scandium tantalate (PST, *ε*_r_ 8000), the difference is roughly two orders of magnitude. Similarly, its dielectric loss exhibits a temperature‐dependent variation that is relatively similar, and tan*δ* values change from 0.009 to 0.15 at different frequencies (Figure 2d). These temperature‐dependent properties near *T*_c_ suggest a dielectric bistability of **1**. According to the Landau theory of phase transitions, dielectric bistability is an indication of improper ferroelectrics, for which *P*_s_ is a second‐order parameter. In contrast to the proper ferroelectrics, the peak *ε*_r_ values at *T*_c_ were at least one order of magnitude higher than the peak off *T*_c_. Hence, pyroelectric FOMs are depressed or attenuated, as indicated in amantadine formate and Sr_0.5_Ba_0.5_Nb_2_O_6_ ;^[^ ^40^, ^41^ ^]^ however, they achieve high FOMs in the vicinity of *T*_c_ for proper ferroelectrics. However, as a pyroelectric candidate, the unusual dielectric bistability of **1** results in a low level of thermal noise and a high detection rate. In this context, dielectric bistability offers the possibility of obtaining large pyroelectric FOMs for use in high‐performance pyroelectric photodetectors of the molecular perovskite‐type ferroelectric family.

### 2.3 Ferroelectric and Pyroelectric Properties

**Figure 3:**
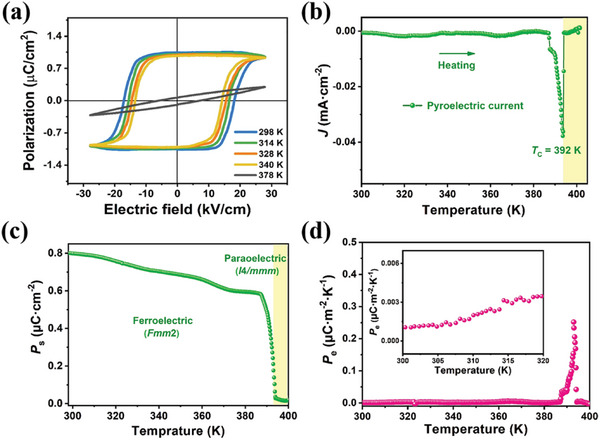
Ferroelectric and pyroelectric properties of **1**. a) *P–E* hysteresis loops obtained at different temperatures. b) Pyroelectric currents with a sharp peak upon heating. c) Temperature dependence of *P*_s_ determined by integrating pyroelectric currents. d) The variable temperature of pyroelectric coefficient (*p*_e_) trace. Inset: The near‐room‐temperature *p*_e_ results.

*P*_s_ versus electric field (*P*–*E*) hysteresis loops provide convincing evidence of ferroelectric materials. Here, typical nonlinear *P*–*E* loops of **1** were recorded to confirm the ferroelectricity along different crystallographic directions. **Figure** **3**a shows the *P*–*E* hysteresis loops obtained by the double‐wave method, giving a saturated *P*_s_ and coercive field (*E*_c_) of ≈1.1 µC cm^−2^ and ≈17 kV cm^−1^ at room temperature, respectively. Rectangular *P*–*E* hysteresis loops were observed along the *c*‐axis and *a*‐axis directions, confirming the biaxial ferroelectricity of **1** (Figure S5, Supporting Information). With the temperature rising above *T*_c_, however, the polarization disappears completely, and the linear *P*–*E* curve confirms the paraelectric state. Subsequently, the pyroelectric properties of **1** were investigated using single crystals. Figure 3b depicts the sharp pyroelectric current generated to compensate for the charge displacement around *T*_c_. The temperature dependence of *P*_s_ obtained from the pyroelectric trace is depicted in Figure 3c, and the *P*_s_ value of 0.8 µC cm^−2^ coincides with that derived from the *P*–*E* trace. According to the formula *p*_e_ = *J*/d*t*/d*T*, the maximum *p*_e_ value of ≈2500 µC m^−2^ K^−1^ is estimated near the *T*_c_, which is comparable to cutting‐edge pyroelectric materials, such as TGS (*p*_e_ = ≈550 µC m^−2^ K^−1^) and Ba_0.85_Ca_0.15_Zr_0.1_Ti_0.9_O_3_ (BZT, *p*_e_ = ≈980 µC m^−2^ K^−1^), but exceeds ferroelectric polymers of PVDF (*p*_e_ = ≈12 µC m^−2^ K^−1^).^[^ ^7^, ^16^, ^42^, ^43^ ^]^

**Figure 4:**
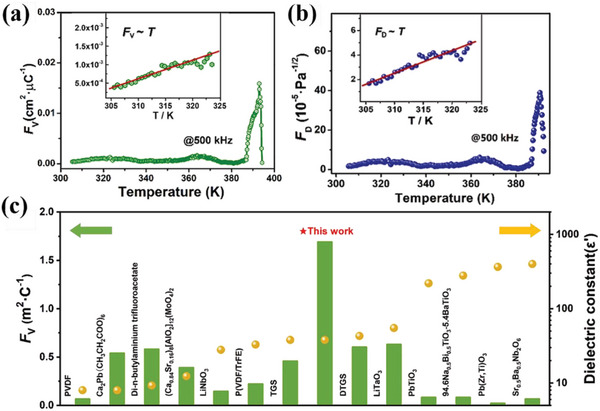
Pyroelectric behaviors and related FOMs of **1**. a) Temperature dependence of *F*_V_ results obtained at *f* = 500 kHz. Insert: The fitting of *F*_V_ ∼ *T* trace. b) Variation of *F*_D_ values at different temperatures. Inset: The fitting of *F*_D_ ∼ *T* curve. c) Comparison of *F*_V_ and *F*_D_ values as a function of dielectric constants in literature and this work.

Notably, the *p*_e_ of **1** exhibited temperature‐dependent behavior, with peak results observed around its *T*_c_ (Figure 3d). The direct measurements of the near‐room‐temperature *p*_e_ values agreed well with the simulated traces (inset of Figure 3d). Considering the bistability of the two dielectric plateaus with almost temperature‐independent characteristics, the dielectric constants of **1** are several orders of magnitude lower than those of normal ferroelectrics, such as 0.7PMN‐0.3PT (*ε*_r_ > 3100) and lead zirconate titanate (PZT, *ε*_r_ > 300).^[^ ^44^, ^45^ ^]^ Consequently, the pyroelectric FOMs of **1** exhibit a similar tendency as a function of temperature. At room temperature, the respective FOMs of *F*_V_ and *F*_D_ were estimated to be ≈5.0×10^−4^ cm^2^ µC^−1^ and ≈2.1×10^−5^ Pa^−1/2^, which are on par with those of the inorganic oxide PbZrO_3_ (*F*_V_ = 6.2×10^−2^ cm^2^ µC^−1^, *F*_D_ = 5.8×10^−5^ Pa^−1/2^). However, with increasing temperature, their respective maxima were greatly enhanced to ≈1.7×10^−2^ cm^2^ µC^−1^ and ≈39×10^−5^ Pa^−1/2^ near *T*_c_ (**Figure** **4**a,b). These figures are higher than those of current state‐of‐the‐art pyroelectric materials. For example, the *F*_V_ peak of **1** is almost three to four times greater than those of DTGS and TGS ^[^ ^16^ ^]^ and more than one order of magnitude higher than those of their inorganic oxide counterparts (Figure 4c). In addition, the *F*_D_ value is two orders of magnitude greater than those of Pb(Zr, Ti)O_3_ (*F*_D_ = 0.4×10^−5^ Pa^−1/2^)^[^ ^46^ ^]^ and PVDF (*F*_D_ = 0.35×10^−5^ Pa^−1/2^) ^[^ ^47^ ^]^ (Figure S6, Supporting Information). These two figure‐or‐merit values were confirmed by light‐induced pyroelectric measurements (Figure 4). In terms of the inferior *p*_e_ values of **1**, the FOMs of improper ferroelectrics are still comparable to those of their proper counterparts, owing to their bistable dielectric properties with relatively small dielectric constants. Our findings reveal that improper ferroelectric materials with dielectric bistability may be suitable pyroelectric contenders for special device applications, such as small‐area (point) thermal imaging and detection.

### 2.4 Broadband Photopyroelectric Effects

**Figure 5:**
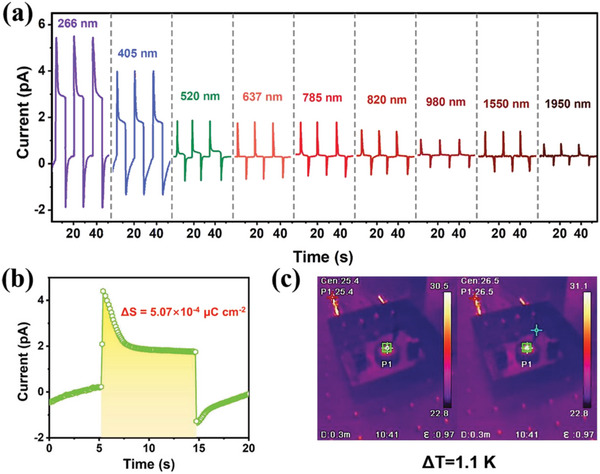
Pyroelectric‐induced photodetection properties of **1**. a) Photoresistor currents measured at various laser illumination wavelengths (100 mW cm^−2^, at zero bias). b) Schematic diagram of the temperature‐up section (the integral area shaded in yellow is 5.07 × 10^−4^ µC cm^−2^) during one on–off response cycle under 405 nm laser illumination. c) Temperature variation of Δ*T* = 1.1 K during the light‐induced pyroelectric measurement.

These studies reveal that the dielectric bistability of **1** plays an important role in its intriguing pyroelectric effects. Because this process depends on the thermally induced variation in *P*_s_, we expect that its crystal‐based photodetector will allow broadband responses in the UV‐to‐NIR‐II spectral range. In common photoelectric devices, it is impossible to directly excite free carriers and generate photocurrents via long‐wave illumination beyond the optical bandgap. Broadband photosensitive materials usually require bandgap modulation through complex chemical doping and the construction of heterojunctions or superlattices.^[^ ^48^ ^]^ Therefore, these materials require complicated fabrication and their operational spectral range is usually fixed. Another potential strategy is based on optical nonlinearity, such as the excitation of carriers through two‐photon^[^ ^49^, ^50^ ^]^ or multiphoton ^[^ ^51^, ^52^, ^53^ ^]^ absorption under high‐intensity laser irradiation (e.g., femtosecond laser pulses), resulting in detectable photocurrents. In contrast, the pyroelectric effect can still generate pyroelectric potentials under long‐wave laser illumination beyond the optical bandgap, and an instantaneous output current is readily achieved under modest light intensities.^[^ ^54^ ^]^ The advantage of this *P*_s_‐directed process is that it facilitates broadband photoactivity. **Figure** **5**a shows the broadband photocurrents produced by crystal‐based detectors of **1** when exposed to laser light with a wavelength range of 266–1950 nm, covering a broad spectrum from the UV to the NIR‐II. Under the same light intensity, the currents behave differently depending on the wavelength. The *I*–*t* curves of **1** under repeated light on/off cycles under 405 nm laser illumination with zero bias are shown in Figure S7 (Supporting Information). At zero bias, the peak pyroelectric current continues to monotonically increase as the incident power increases because of the increased number of photons, accelerated rate of temperature rise, and creation of electron–hole pairs.^[^ ^54^, ^55^, ^56^, ^57^ ^]^ The peak pyroelectric current continued to increase as the power density increased. The photothermal release characteristics of **1** were quantified using a dynamic approach with continuous temperature changes.^[^ ^5^, ^58^ ^]^ We analyzed the thermal equilibrium process of the device under illumination. During one on–off response cycle under 405 nm laser illumination, the integral area outlined in yellow on the schematic diagram of the temperature‐up section is 5.07×10^−4^ µC cm^−2^ K^−1^, as presented in Figure 5b. As measured using thermal imaging, the identifiable temperature change in the sample was 1.1 K. At 100 mW cm^−2^, the *p*_e_ was estimated to be 4.61×10^−3^ µC cm^−2^ K^−1^ (Figure 5c). Its photothermal release properties were evaluated using hysteresis measurements under the same temperature variations (called the static method).^[^ ^5^ ^]^ The obtained value from the *P*_s_–*T* curve is 3.55×10^−3^ µC cm^−2^ K^−1^ (known as the hysteresis loop measurement, Figure S8, Supporting Information), which coincides with that deduced from the dynamic method, illustrating a high photothermal conversion efficiency of **1**. These findings reveal the potential of improper ferroelectrics as new self‐powered broadband candidates through the pyroelectric effect and offer a promising future for the assembly of high‐performance infrared detectors. The phase stability of **1** was evaluated after exposure to environmental conditions for 30 d. The measured powder X‐ray diffraction pattern was consistent with the simulated result (Figure S9, Supporting Information), and the photopyroelectric behavior of **1** showed no significant attenuation under 405 nm laser irradiation (≈100 mW cm^−2^, Figure S10, Supporting Information). These results revealed the notable phase and photoactive stability of **1**.

## 3 Conclusion

In summary, we successfully exploited a perovskite‐type hybrid ferroelectric by confining EA in cavities as (IA)_2_(EA)_2_Pb_3_Cl_10_, which showed bistable dielectric properties and biaxial ferroelectricity. Most notably, the pyroelectric FOMs exhibited a significant enhancement in the vicinity of *T*_c_, for which the *F*_V_ and *F*_D_ peak values were far beyond those of their normal ferroelectric counterparts. These findings are closely related to its dielectric bistability, revealing its potential for achieving an efficient signal‐to‐noise ratio for detecting infrared/thermal radiation. Furthermore, the photoinduced pyroelectric effect in **1** enabled a broadband optical response, covering the spectral range from the UV to NIR‐II (266−1950 nm). This study revealed that ferroelectric materials with dielectric bistability are promising pyroelectric candidates for certain special device applications, such as small‐area thermal detectors.^[^ ^59^ ^]^

## Conflict of Interest

The authors declare no conflict of interest.

## Supporting information

Supporting Information

Supporting cif files

## Data Availability

Research data are not shared.

## References

[1] Z. Liu , T. Lu , X. Dong , G. Wang , Y. Liu , IEEE Trans. Ultrason. Ferroelectr. Freq. Control 2021, 68, 242. 32956053 10.1109/TUFFC.2020.3025168

[2] Y. Zhang , P. T. T. Phuong , E. Roake , H. Khanbareh , Y. Wang , S. Dunn , C. Bowen , Joule 2020, 4, 301.

[3] C. R. Bowen , J. Taylor , E. LeBoulbar , D. Zabek , A. Chauhan , R. Vaish , Energy Environ. Sci. 2014, 7, 3836.

[4] P. Guggilla , A. K. Batra , J. R. Currie , M. D. Aggarwal , M. A. Alim , R. B. Lal , Mater. Lett. 2006, 60, 1937.

[5] S. Jachalke , E. Mehner , H. Stöcker , J. Hanzig , M. Sonntag , T. Weigel , T. Leisegang , D. C. Meyer , Appl. Phys. Rev. 2017, 4, 021303.

[6] W. Li , G. Tang , G. Zhang , H. M. Jafri , J. Zhou , D. Liu , Y. Liu , J. Wang , K. Jin , Y. Hu , H. Gu , Z. Wang , J. Hong , H. Huang , L. Chen , S. Jiang , Q. Wang , Sci. Adv. 2021, 7, eabe3068. 33514555 10.1126/sciadv.abe3068 PMC7846162

[7] R. W. Whatmore , Rep. Prog. Phys. 1986, 49, 1335.

[8] V. Dvořák , Ferroelectrics 1974, 7, 1.

[9] N. A. Benedek , C. J. Fennie , Phys. Rev. Lett. 2011, 106, 107204. 21469829 10.1103/PhysRevLett.106.107204

[10] Z. Sun , Y. Tang , S. Zhang , C. Ji , T. Chen , J. Luo , Adv. Mater. 2015, 27, 4795. 26179321 10.1002/adma.201501923

[11] Y. Ma , J. Wang , W. Guo , S. Han , J. Xu , Y. Liu , L. Lu , Z. Xie , J. Luo , Z. Sun , Adv. Funct. Mater. 2021, 31, 2103012.

[12] K. S. Srikanth , R. Vaish , J. Eur. Ceram. Soc. 2017, 37, 3927;

[13] S.‐M. Zeng , X.‐G. Tang , Q.‐X. Liu , Y.‐P. Jiang , M.‐D. Li , W.‐H. Li , Z.‐H. Tang , J. Alloys Compd. 2019, 776, 731.

[14] K. Iijima , S. Kawashima , I. Ueda , Jpn. J. Appl. Phys. 1985, 24, 482.

[15] A. M. Glass , Phys. Rev. 1968, 172, 564.

[16] P. Felix , P. Gamot , P. Lacheau , Y. Raverdy , Ferroelectrics 1977, 17, 543.

[17] Y. Bai , P. Tofel , J. Palosaari , H. Jantunen , J. Juuti , Adv. Mater. 2017, 29, 1700767. 10.1002/adma.201700767 28585344

[18] A. Berenov , P. Petrov , B. Moffat , J. Phair , L. Allers , R. W. Whatmore , APL Mater. 2021, 9, 041108.

[19] C. Montero‐Tavera , M. D. Durruthy‐Rodríguez , F. D. Cortés‐Vega , J. M. Yañez‐Limón , J. Adv. Cerams 2020, 9, 329.

[20] A. Shaulov , W. A. Smith , G. M. Loiacono , M. I. Bell , Y. H. Tsuo , Ferroelectrics 2011, 27, 117.

[21] X.‐J. Song , T. Zhang , Z.‐X. Gu , Z.‐X. Zhang , D.‐W. Fu , X.‐G. Chen , H.‐Y. Zhang , R.‐G. Xiong , J. Am. Chem. Soc. 2021, 143, 5091. 33755474 10.1021/jacs.1c00613

[22] H. L. B. Boström , M. S. Senn , A. L. Goodwin , Nat. Commun. 2018, 9, 2380. 29915202 10.1038/s41467-018-04764-x PMC6006342

[23] L. Tang , S. Han , Y. Ma , Y. Liu , L. Hua , H. Xu , W. Guo , B. Wang , Z. Sun , J. Luo , Chem. Mater. 2022, 34, 8898.

[24] P. Peksa , A. Nowok , F. Formalik , J. K. Zaręba , J. Trzmiel , A. Gągor , M. Mączka , A. Sieradzki , J. Mater. Chem. C 2022, 10, 6866.

[25] A. Sieradzki , M. Mączka , M. Simenas , J. K. Zaręba , A. Gągor , S. Balciunas , M. Kinka , A. Ciupa , M. Nyk , V. Samulionis , J. Banys , M. Paluch , S. Pawlus , J. Mater. Chem. C 2018, 6, 9420.

[26] Y. Zhang , H.‐Y. Ye , H.‐L. Cai , D.‐W. Fu , Q. Ye , W. Zhang , Q. Zhou , J. Wang , G.‐L. Yuan , R.‐G. Xiong , Adv. Mater. 2014, 26, 4515. 24789577 10.1002/adma.201400806

[27] J. A. Hill , A. L. Thompson , A. L. Goodwin , J. Am. Chem. Soc. 2016, 138, 5886. 27057759 10.1021/jacs.5b13446 PMC4894656

[28] S. G. Duyker , J. A. Hill , C. J. Howard , A. L. Goodwin , J. Am. Chem. Soc. 2016, 138, 11121. 27533044 10.1021/jacs.6b06785

[29] H. L. B. Boström , J. A. Hill , A. L. Goodwin , Phys. Chem. Chem. Phys. 2016, 18, 31881. 27841402 10.1039/c6cp05730f

[30] W. Zhang , Y. Cai , R.‐G. Xiong , H. Yoshikawa , K. Awaga , Angew. Chem., Int. Ed. 2010, 49, 6608. 10.1002/anie.201001208 20715229

[31] N. L. Evans , P. M. M. Thygesen , H. L. B. Boström , E. M. Reynolds , I. E. Collings , A. E. Phillips , A. L. Goodwin , J. Am. Chem. Soc. 2016, 138, 9393. 27414161 10.1021/jacs.6b05208

[32] S. Wang , X, L. , L. Li , C. Ji , Z. Sun , Z. Wu , M. Hong , J. Luo , J. Am. Chem. Soc. 2019, 141, 7693. 31046266 10.1021/jacs.9b02558

[33] S. Wang , L. Li , W. Weng , C. Ji , X. Liu , Z. Sun , W. Lin , M. Hong , J. Luo , J. Am. Chem. Soc. 2020, 142, 55. 31841326 10.1021/jacs.9b10919

[34] K. Aizu , J. Phys. Soc. Jpn. 1969, 27, 387.

[35] P.‐P. Shi , Y.‐Y. Tang , P.‐F. Li , W.‐Q. Liao , Z.‐X. Wang , Q. Ye , R.‐G. Xiong , Chem. Soc. Rev. 2016, 45, 3811. 27051889 10.1039/c5cs00308c

[36] X. Liu , Z. Xu , P. Long , Y. Yao , C. Ji , L. Li , Z. Sun , M. Hong , J. Luo , Chem. Mater. 2020, 32, 8965.

[37] S. Han , M. Li , Y. Liu , W. Guo , M.‐ Hong , Z. Sun , J. Luo , Nat. Commun. 2021, 12, 284. 33436587 10.1038/s41467-020-20530-4 PMC7804191

[38] Y.‐Y. Tang , W.‐Y. Zhang , P.‐F. Li , H.‐Y. Ye , Y.‐M. You , R.‐G. Xiong , J. Am. Chem. Soc. 2016, 138, 15784. 27934003 10.1021/jacs.6b10595

[39] M. Li , Y. Xu , S. Han , J. Xu , Z. Xie , Y. Liu , Z. Xu , M. Hong , J. Luo , Z. Sun , Adv. Mater. 2020, 32, 2002972. 10.1002/adma.202002972 32705717

[40] J. Zhou , S. Jin , C. Chai , M. Hao , X. Zhong , T. Ying , J. Guo , X. Chen , Innovation 2020, 3, 100204. 10.1016/j.xinn.2021.100204 PMC8803662 35128503

[41] M. Venet , I. Santos , J. Eiras , D. Garcia , Solid State Ionics 2006, 177, 589.

[42] M. Sharma , V. P. Singh , S. Singh , P. Azad , B. Ilahi , N. A. Madhar , J. Electron. Mater. 2018, 47, 4882.

[43] S. Patel , A. Chauhan , R. Vaish , Solid State Sci. 2016, 52, 10.

[44] R. Chukka , J. Cheah , Z. Chen , P. Yang , S. Shannigrahi , J. Wang , L. Chen , Appl. Phys. Lett. 2011, 98, 242902.

[45] X. Wang , Z. Gong , Y. Tang , X. He , Q. He , Z. Peng , D. Sun , Ferroelectrics 2010, 403, 191.

[46] R. W. Whatmore , J. Electroceram. 2004, 13, 139.

[47] A. Navid , C. S. Lynch , L. Pilon , Smart Mater. Struct. 2010, 19, 055006.

[48] H. Kim , S. Z. Uddin , D.‐H. Lien , M. Yeh , N. S. Azar , S. Balendhran , T. Kim , N. Gupta , Y. Rho , C. P. Grigoropoulos , K. B. Crozier , A. Javey , Nature 2021, 596, 232. 34381234 10.1038/s41586-021-03701-1

[49] A. D. Bristow , N. Rotenberg , H. M. van Driel , Appl. Phys. Lett. 2007, 90, 191104.

[50] M. Reichert , A. L. Smirl , G. Salamo , D. J. Hagan , E. W. V. Stryland , Phys. Rev. Lett. 2016, 117, 073602. 27563962 10.1103/PhysRevLett.117.073602

[51] W. C. Hurlbut , Y.‐S. Lee , K. Vodopyanov , P. Kuo , M. Fejer , Opt. Lett. 2007, 32, 668. 17308596 10.1364/ol.32.000668

[52] S. Pearl , N. Rotenberg , H. M. van Driel , Appl. Phys. Lett. 2008, 93, 131102.

[53] A. Nevet , A. Hayat , M. Orenstein , Opt. Lett. 2011, 36, 725. 21368962 10.1364/OL.36.000725

[54] W. Guo , H. Xu , W. Weng , L. Tang , Y. Ma , Y. Liu , L. Hua , B. Wang , J. Luo , Z. Sun , Angew. Chem., Int. Ed. 2022, 134, e202213477. 10.1002/anie.202213477 36326079

[55] Z. Yang , X. Li , L. Gao , W. Zhang , X. Wang , H. Liu , S. Wang , C. Pan , L. Guo , Nano Energy 2022, 98, 107312.

[56] K. Zhao , B. Ouyang , C. R. Bowen , Y. Yang , Nano Energy 2022, 77, 105152.

[57] L. Guo , Y. Qi , Z. Yang , L. Zhao , W. Zhang , X. Wang , H. Liu , G. Yan , S. Wang , C. Pan , Nano Energy 2022, 102, 107714.

[58] A. G. Chynoweth , J. Appl. Phys. 1956, 27, 78.

[59] R. Pandey , G. Vats , J. Yun , C. R. Bowen , A. W. Y. Ho‐Baillie , J. Seidel , K. T. Butler , S. I. Seok , Adv. Mater. 2019, 31, 1807376. 10.1002/adma.201807376 31441161

