# Supplementary material for: Improper High‐T c Perovskite Ferroelectric with Dielectric Bistability Enables Broadband Ultraviolet‐to‐Infrared Photopyroelectric Effects
Source: Adv Sci (Weinh). 2023 Apr 23;10(19):2301064. doi: 10.1002/advs.202301064 (PMC10323668; doi:10.1002/advs.202301064)
Supplement: Supplementary file 1 — Supporting Information [file ADVS-10-2301064-s002.pdf]

ADVANCED SCIENCE
Open Access

Supporting Information

for *Adv. Sci.*, DOI 10.1002/advs.202301064

Improper High-T_c Perovskite Ferroelectric with Dielectric Bistability Enables Broadband Ultraviolet-to-Infrared Photopyroelectric Effects

Lina Hua, Jiaqi Wang, Yi Liu, Wuqian Guo, Yu Ma, Haojie Xu, Shiguo Han, Junhua Luo and Zhihua Sun**

Supporting Information

Improper High-Tc Perovskite Ferroelectric with Dielectric Bistability Enables Broadband Ultraviolet-to-Infrared Photopyroelectric Effects

Lina Hua, Jiaqi Wang, Yi Liu, Wuqian Guo, Yu Ma, Haojie Xu, Shiguo Han, Junhua Luo, and Zhihua Sun**

Experimental Section

Synthesis and single-crystal growth. All the chemical reagents were purchased commercially and used without any further purification. Compound **1** was prepared by mixing a stoichiometric ratio of $Pb(COOH)_2 \cdot 3H_2O$ (2.0 g, 0.005 mol), isoamylamine (0.28 g, 0.003 mol) and ethylamine (1.68 g, 0.04 mol). The appropriate chemical reagents were gradually introduced to the hydrochloric acid solution during the synthesis process. The mixture was then heated to boiling while being constantly stirred. By using the temperature cooling method at room temperature, crystals were produced.

Single-Crystal structure determination. We recorded X-ray diffraction data using the Mo *K*α radiation (λ = 0.77 Å) and the Bruker D8 diffractometer. The structures were resolved by the direct method, and the *SHELXTL* software packing was used to enhance the full-matrix least-squares results on F^2. While all H atoms were created geometrically and refined using a "riding" model with *Uiso* = 1.2 *Ueq* (C), all non-H atoms were refined anisotropically (C). The *Olex*2 software was used to solve and refine the aforementioned structure. Crystal data for **1** at 300 and 400 K are listed in Table S1.

Characterization. Powder X-ray diffractometry (PXRD) data were recorded on the MiniFlex 600 X-ray diffractometer equipped with Cu *K*α radiation. The NETZSCH DSC 200 F3 was used for differential scanning calorimetry (DSC) and c_p studies of **1** with heating/cooling rates of 10 K/min in a N_2 environment. To measure the dielectric constant, single crystals of type **1** with a silver paste-coated surface were utilized. The dielectric analyses were performed on the TongHui TH2828 analyzer. The laser used in the SHG measurement was an OPOTEK Vibrant 355 II, and the instrument used was the FLS 920 from Edinburgh Instruments. A lateral structural device was equipped with a Keithley 6517B source meter. A Linkam TS1500 heating stage was used to regulate the temperature during the measurements. The light lighting was accomplished using THORLABS pigtailed laser diodes (LP405-

MF300, LP520-MF100, LP637-SF70, LP785-SF100, and LP940-SF30). A single crystal's flat surface was coated with two symmetric Au electrodes. It was established that the electrode materials had no discernible impact on the photo-pyroelectric characteristics. The New Focus 1550nm and home-made 1950nm lasers (Velocity TLB-6730, LDM-1950-20) were utilized for the infrared light illumination, while the handmade 266nm laser (LDMQ-266-10) was employed for the ultraviolet light lighting. System for thermal imaging: HIKVISION H21pro. Using a Keithley 6517B electrometer and a heating procedure with a consistent heating rate, the pyroelectric current was recorded. The Chynoweth method was used to determine the dynamic of pyroelectric current at room temperature. A pulsed laser modulated at a low frequency of 0.05 Hz was used to modify the sample's temperature periodically.

[CCDC 2240579 and 2240580 contain the supplementary crystallographic data for single-crystal structures of ferroelectric (300 K) and paraelectric phase (400 K), respectively. These data can be obtained free of charge from The Cambridge Crystallographic Data Centre via www.ccdc.cam.ac.uk/data_request/cif.]

Figures

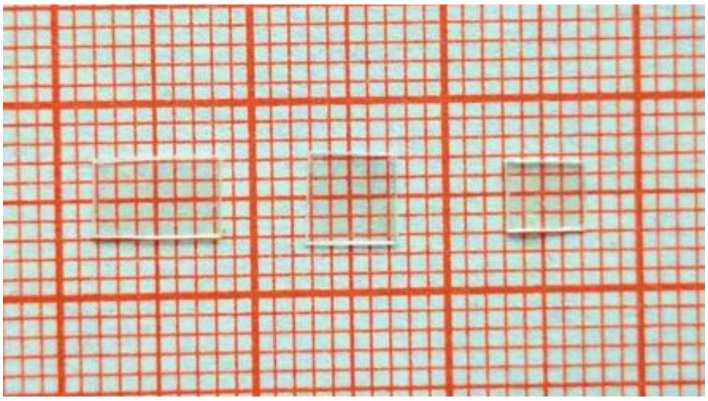

Figure S1. As-grown bulk single-crystal of **1**.

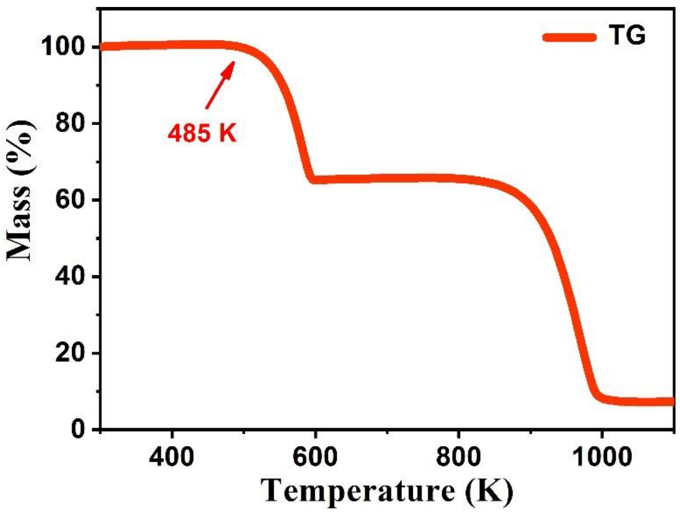

Figure S2. Thermal analysis of **1**. The result indicates that it has thermal stability up to 485K, much higher than its T_c at 392 K.

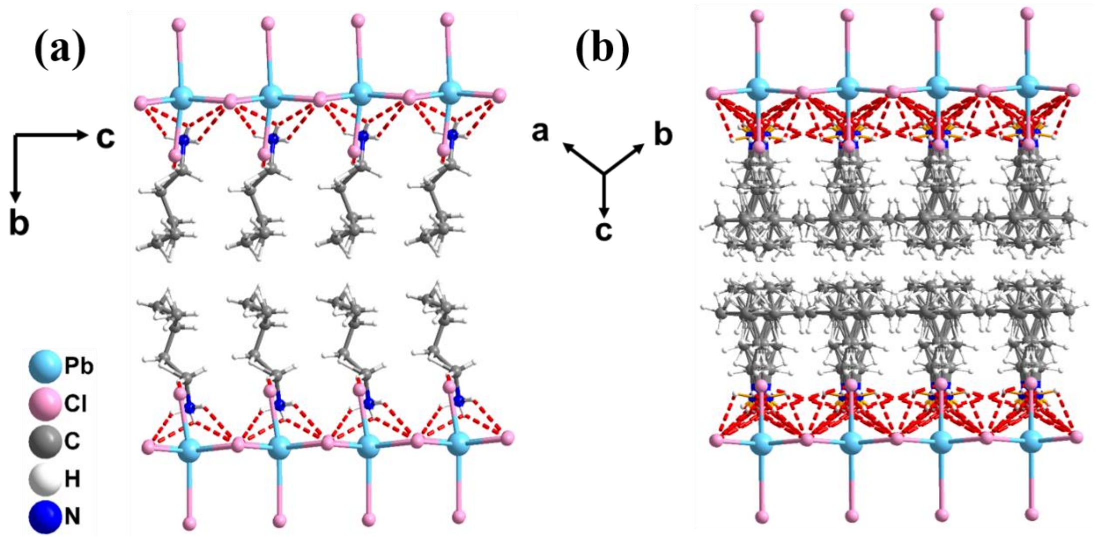

Figure S3. The N-H···Cl hydrogen-bonding interactions between organic cations and inorganic perovskite frameworks of **1** at 300 (a) and 400 K (b), respectively. Red dotted lines represent the N-H···Cl hydrogen bonds.

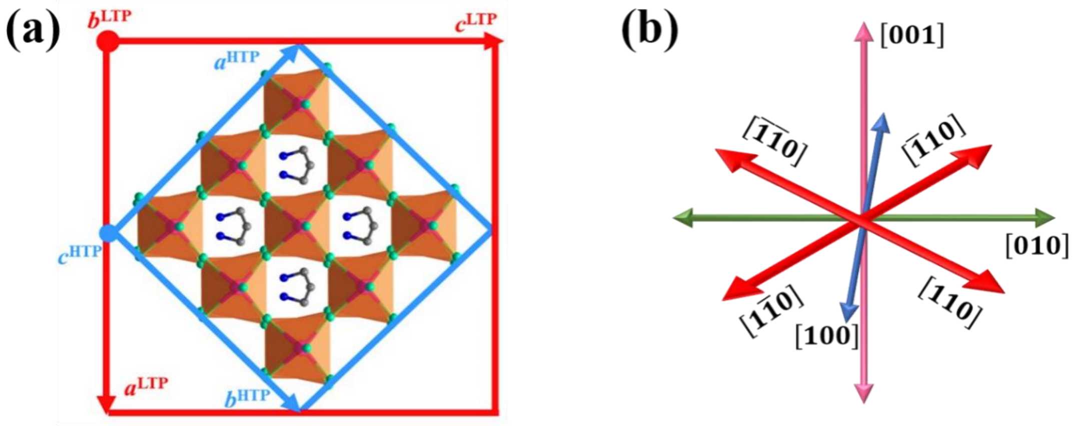

Figure S4. Temperature-induced transformation of crystallographic. (a) Change of unit cells from FEP (red) to PEP (blue). (b) Diagram of four equivalent [110] directions (red arrowheads) at PEP that coincides with the polar [001] direction at FEP. This reveals the biaxial ferroelectricity.

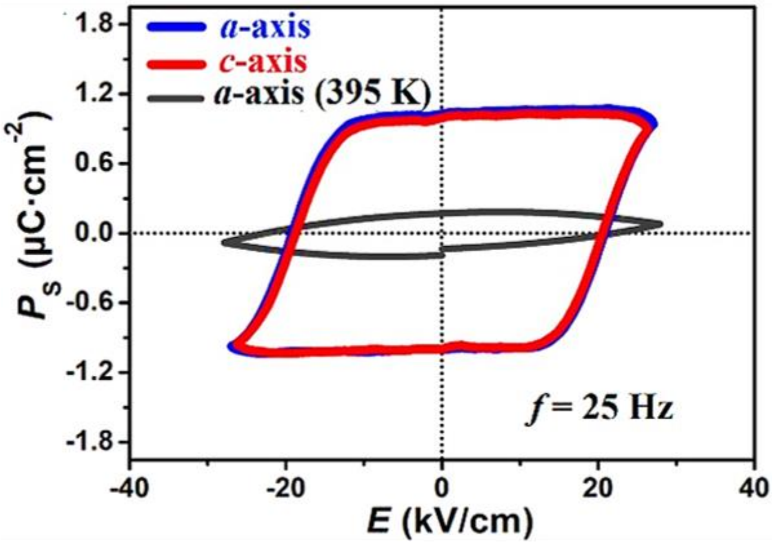

Figure S5. The *P-E* hysteresis loops were measured along the *a*- and *c*- axis crystal directions at room temperature and 395 K, respectively.

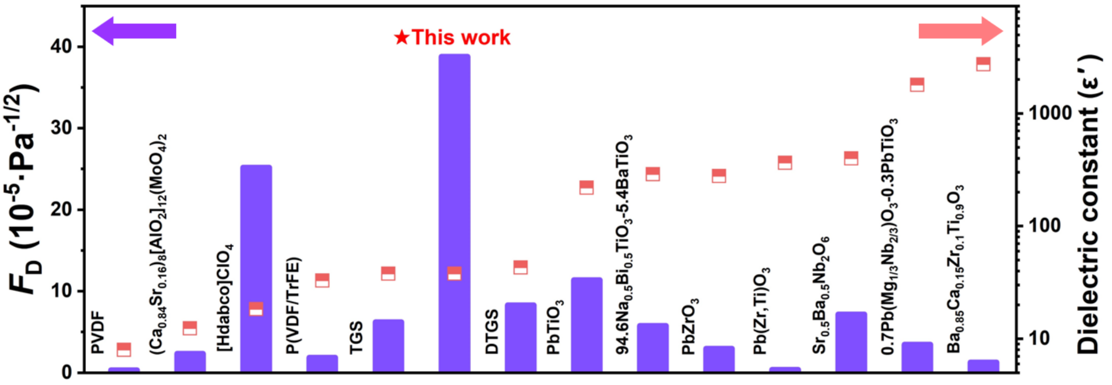

Figure S6. Comparison of F_D values as a function of dielectric constants in some literature and this work.

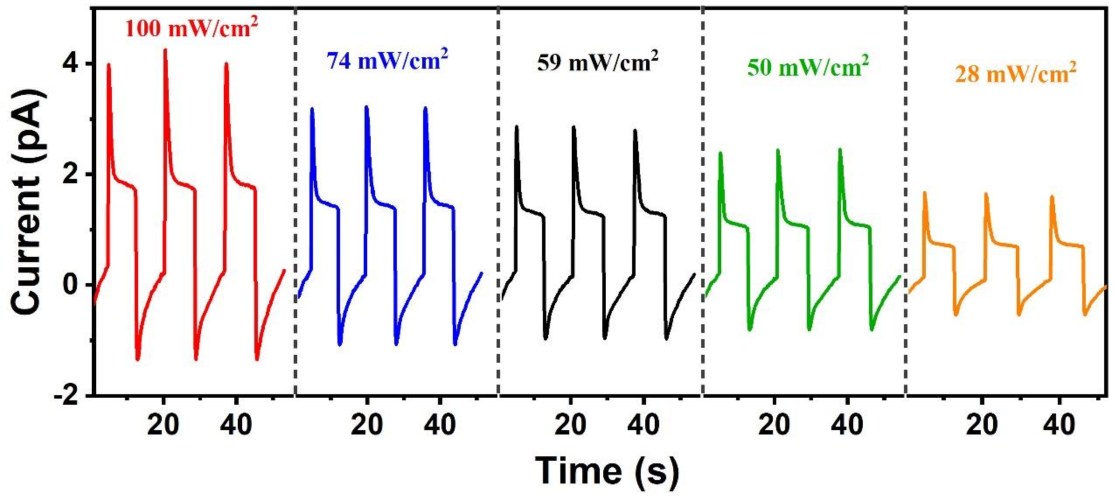

Figure S7. Variation of photocurrent intensities as a function of light density at 405 nm illumination.

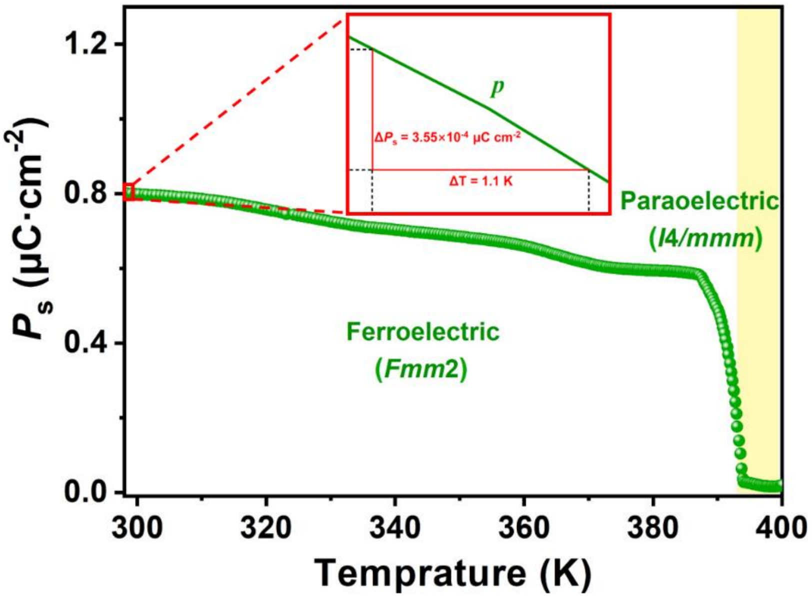

Figure S8. The slope of the temperature-dependent curve of P_s, defining the pyroelectric coefficient p_e (see the inset).

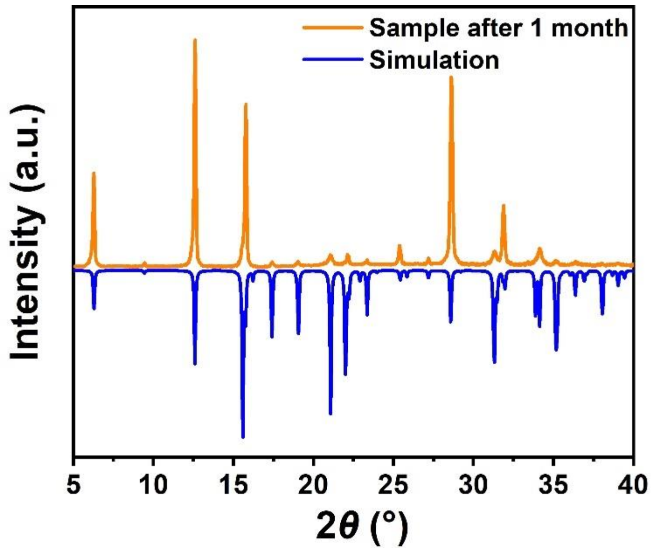

Figure S9. Powder X-ray diffraction patterns of **1** recorded on the sample exposed to air condition after 1 month.

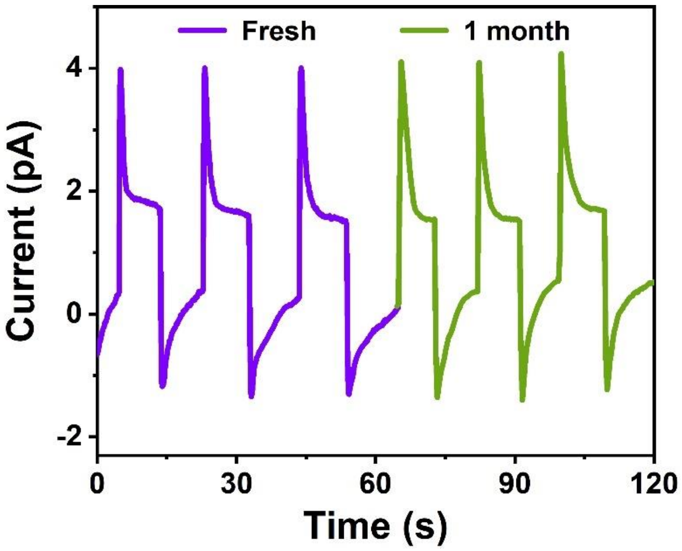

Figure S10. Photopyroelectric behaviors of **1** were measured on the fresh crystal sample and after exposure to air conditions.

Tables

Table S1. Crystal data for **1** collected at low-temperature phase (LTP, 300K) and high-temperature phase (HTP, 400 K), respectively.

Empirical formula	$C_{14}H_{44}Cl_{10}N_4Pb_3$	$C_{14}H_{44}Cl_{10}N_4Pb_3$
Formula weight	1244.60	1244.60
Temperature/K	300	400
Crystal system	orthorhombic	tetragonal
Space group	*Fmm*2	*I*4/*mmm*
a/Å	8.0787(15)	5.7493(3)
b/Å	56.156(10)	5.7493(3)
c/Å	8.0644(15)	56.681(5)
α/°	90	90
β/°	90	90
γ/°	90	90
Volume/$Å^3$	3658.6(12)	1873.5(3)
Z	4	2
ρ_{calc} g/cm^3	2.260	2.206
μ/mm^{-1}	14.514	14.171
F(000)	2288.0	1144.0
2Θ range for data collection/°	4.352 to 55.05	7.124 to 55.012
Index ranges	$-10 \leq h \leq 9$, $-72 \leq k \leq 70$, $-10 \leq l \leq 9$	$-7 \leq h \leq 6$, $-7 \leq k \leq 7$, $-73 \leq l \leq 72$
Reflections collected	7784	5687
Independent reflections	2233 [R_{int}= 0.0614, R_{sigma} = 0.0565]	743 [R_{int}= 0.0579, R_{sigma} = 0.0364]
Data/restraints/parameters	2233/113/112	743/91/90
Goodness-of-fit on F^2	1.072	1.060
Final R indexes [I≥2σ (I)]	R_1 = 0.0670, wR_2 = 0.1705	R_1 = 0.0367, wR_2 = 0.0970
Final R indexes [all data]	R_1 = 0.0882, wR_2 = 0.1859	R_1 = 0.0540, wR_2 = 0.1080

Table S2. Bond lengths of **1** at 300 K.

Atom	Atom	Length/Å	Atom	Atom	Length/Å
Pb1	Cl3	3.278(4)	Pb2	Cl3^{1}	2.851(4)
Pb1	Cl2	2.755(5)	C7	C6	1.483(15)
Pb1	Cl1	2.594(5)	N1	C1	1.495(12)
Pb2	Cl4^{1}	2.892(6)	C2	C3	1.506(11)
Pb2	Cl4^{2}	2.944(5)	C2	C1	1.514(14)
Pb2	Cl4^{3}	2.944(5)	C3	C5	1.508(9)
Pb2	Cl4	2.892(6)	C3	C4	1.484(14)
Pb2	Cl4	2.851(4)	C6	N7	1.510(16)

Symmetry transformations used to generate equivalent atoms: ^{1}1-X,1-Y,+Z; ^{2}3/2-X,1-Y,-1/2+Z; ^{3}-1/2+X,+Y,-1/2+Z

Table S3. The selected bond angles of crystal **1** at 300 K.

Bond	Angle/°	Bond	Angle/°
Cl2-Pb1-Cl3	93.41(18)	Cl3^{1}-Pb2-Cl3	178.0(5)
Cl1-Pb1-Cl3	171.2(3)	Pb2-Cl4-Pb2^{4}	155.9(2)
Cl1-Pb1-Cl2	80.75(19)	Pb2-Cl3-Pb1	94.53(5)
Cl4^{1}-Pb2-Cl4^{2}	155.9(2)	Pb1-Cl2-Pb1^{3}	174.60(8)
Cl4-Pb2-Cl4^{2}	90.12(5)	C3-C2-C1	93.70(5)
Cl4-Pb2-Cl4^{1}	65.8(2)	C2-C3-C5	89.62(3)
Cl4-Pb2-Cl4^{3}	155.9(2)	C4-C3-C2	87.61(6)
Cl4^{3}-Pb2-Cl4^{2}	113.9(2)	C4-C3-C5	175.85(6)
Cl3-Pb2-Cl4^{3}	90.54(14)	N1-C1-C2	93.32(6)
Cl3^{1}-Pb2-Cl4^{1}	89.2(2)	C7-C6-N7	111.8(13)
Cl3-Pb2-Cl4^{2}	90.54(14)		

Symmetry transformations used to generate equivalent atoms: ^{1}1-X,1-Y,+Z; ^{2}3/2-X,1-Y,-1/2+Z; 3-1/2+X,+Y,-1/2+Z; ^{4}1/2+X,+Y,1/2+Z

Table S4. Hydrogen bonds of crystal **1** at 300 K.

**D-H**	**d(D-H)**	**d(H..A)**	**<DHA**	**d(D..A)**	**A**
C2-H2B	0.970	2.961	133.25	3.694	Cl1
C1^a-H1E^a	0.970	2.483	160.45	3.413	Cl1[x-1/2, y, z+1/2]
C6^a-H6A^a	0.970	2.232	154.48	3.135	Cl4 [-x+1/2, -y+1, z-1/2]
C6^a-H6B^a	0.970	2.188	145.30	3.037	Cl3 [x-1, y, z]
N7^a-H7E^a	0.890	2.444	118.88	2.977	Cl4 [x-1, y, z]
N7^a-H7F^a	0.890	2.555	117.88	3.074	Cl3 [x-1/2, y, z+1/2]
