# Supplementary material for: Improper High‐T c Perovskite Ferroelectric with Dielectric Bistability Enables Broadband Ultraviolet‐to‐Infrared Photopyroelectric Effects
Source: Adv Sci (Weinh). 2023 Apr 23;10(19):2301064. doi: 10.1002/advs.202301064 (PMC10323668; doi:10.1002/advs.202301064)

checkCIF/PLATON report

You have not supplied any structure factors. As a result the full set of tests cannot be run.

THIS REPORT IS FOR GUIDANCE ONLY. IF USED AS PART OF A REVIEW PROCEDURE FOR PUBLICATION, IT SHOULD NOT REPLACE THE EXPERTISE OF AN EXPERIENCED CRYSTALLOGRAPHIC REFEREE.

No syntax errors found. CIF dictionary Interpreting this report

Datablock: w03300K

Bond precision: C-C = 0.0120 A Wavelength=0.71073

Cell:	a=8.0787(15)	b=56.156(10)	c=8.0644(15)
	alpha=90	beta=90	gamma=90
Temperature:	300 K		

	Calculated	Reported
Volume	3658.6(12)	3658.6(12)
Space group	F m m 2	F m m 2
Hall group	F 2 -2	F 2 -2
Moiety formula	2(Cl5 Pb1.50), 2(C5 H14 N), 2(C2 H8 N)	0.5(Cl20 Pb6), C4 H16 N2, C10 H28 N2
Sum formula	C14 H44 Cl10 N4 Pb3	C14 H44 Cl10 N4 Pb3
Mr	1244.63	1244.60
Dx,g cm-3	2.260	2.260
Z	4	4
Mu (mm-1)	14.513	14.514
F000	2288.0	2288.0
F000'	2256.38	
h,k,lmax	10,72,10	10,72,10
Nref	2304[1238]	2233
Tmin,Tmax		0.004,0.021
Tmin'		

Correction method= # Reported T Limits: Tmin=0.004 Tmax=0.021
AbsCorr = NONE

Data completeness= 1.80/0.97 Theta(max)= 27.525

R(reflections)= 0.0670(1682) wR2(reflections)= 0.1858(2233)

S = 1.072 Npar= 112

The following ALERTS were generated. Each ALERT has the format
test-name_ALERT_alert-type_alert-level.
Click on the hyperlinks for more details of the test.

Alert level B

```
PLAT242_ALERT_2_B Low    'MainMol' Ueq as Compared to Neighbors of        Pb1 Check
```

Alert level C

```
STRVA01_ALERT_4_C           Flack test results are ambiguous.
          From the CIF: _refine_ls_abs_structure_Flack    0.500
          From the CIF: _refine_ls_abs_structure_Flack_su   70.000
PLAT042_ALERT_1_C Calc. and Reported MoietyFormula Strings  Differ     Please Check
PLAT053_ALERT_1_C Minimum Crystal Dimension Missing (or Error) ...     Please Check
PLAT054_ALERT_1_C Medium  Crystal Dimension Missing (or Error) ...     Please Check
PLAT055_ALERT_1_C Maximum Crystal Dimension Missing (or Error) ...     Please Check
PLAT094_ALERT_2_C Ratio of Maximum / Minimum Residual Density ....       2.08 Report
PLAT234_ALERT_4_C Large Hirshfeld Difference N1       --C1       .       0.20 Ang.
PLAT241_ALERT_2_C High   'MainMol' Ueq as Compared to Neighbors of        Cl3 Check
PLAT242_ALERT_2_C Low    'MainMol' Ueq as Compared to Neighbors of        Pb2 Check
PLAT260_ALERT_2_C Large Average Ueq of Residue Including       Pb1      0.115 Check
PLAT260_ALERT_2_C Large Average Ueq of Residue Including        N1      0.215 Check
PLAT260_ALERT_2_C Large Average Ueq of Residue Including        N7      0.224 Check
PLAT342_ALERT_3_C Low Bond Precision on  C-C Bonds ...............      0.012 Ang.
```

Alert level G

```
PLAT002_ALERT_2_G Number of Distance or Angle Restraints on AtSite         12 Note
PLAT003_ALERT_2_G Number of Uiso or Uij Restrained non-H Atoms ...          9 Report
PLAT004_ALERT_5_G Polymeric Structure Found with Maximum Dimension          2 Info
PLAT005_ALERT_5_G No Embedded Refinement Details Found  in the CIF     Please Do !
PLAT007_ALERT_5_G Number of Unrefined Donor-H Atoms .............           6 Report
PLAT083_ALERT_2_G SHELXL Second Parameter in WGHT  Unusually Large      92.41 Why ?
PLAT232_ALERT_2_G Hirshfeld Test Diff (M-X)  Pb2      --Cl4      .       10.8 s.u.
PLAT300_ALERT_4_G Atom Site Occupancy of C1         Constrained at        0.5 Check
PLAT300_ALERT_4_G Atom Site Occupancy of C4         Constrained at        0.5 Check
PLAT300_ALERT_4_G Atom Site Occupancy of C5         Constrained at        0.5 Check
PLAT300_ALERT_4_G Atom Site Occupancy of H1A        Constrained at        0.5 Check
PLAT300_ALERT_4_G Atom Site Occupancy of H1B        Constrained at        0.5 Check
PLAT300_ALERT_4_G Atom Site Occupancy of H1C        Constrained at        0.5 Check
PLAT300_ALERT_4_G Atom Site Occupancy of H1D        Constrained at        0.5 Check
PLAT300_ALERT_4_G Atom Site Occupancy of H1E        Constrained at        0.5 Check
PLAT300_ALERT_4_G Atom Site Occupancy of H2A        Constrained at        0.5 Check
PLAT300_ALERT_4_G Atom Site Occupancy of H2B        Constrained at        0.5 Check
PLAT300_ALERT_4_G Atom Site Occupancy of H3         Constrained at        0.5 Check
PLAT300_ALERT_4_G Atom Site Occupancy of H4A        Constrained at        0.5 Check
PLAT300_ALERT_4_G Atom Site Occupancy of H4B        Constrained at        0.5 Check
PLAT300_ALERT_4_G Atom Site Occupancy of H4C        Constrained at        0.5 Check
PLAT300_ALERT_4_G Atom Site Occupancy of H5A        Constrained at        0.5 Check
PLAT300_ALERT_4_G Atom Site Occupancy of H5B        Constrained at        0.5 Check
PLAT300_ALERT_4_G Atom Site Occupancy of H5C        Constrained at        0.5 Check
PLAT300_ALERT_4_G Atom Site Occupancy of N7         Constrained at        0.5 Check
PLAT300_ALERT_4_G Atom Site Occupancy of C6         Constrained at        0.5 Check
PLAT300_ALERT_4_G Atom Site Occupancy of H6A        Constrained at        0.5 Check
```

```
PLAT300_ALERT_4_G Atom Site Occupancy of H6B        Constrained at        0.5 Check
PLAT300_ALERT_4_G Atom Site Occupancy of H7A        Constrained at        0.5 Check
PLAT300_ALERT_4_G Atom Site Occupancy of H7B        Constrained at        0.5 Check
PLAT300_ALERT_4_G Atom Site Occupancy of H7C        Constrained at        0.5 Check
PLAT300_ALERT_4_G Atom Site Occupancy of H7D        Constrained at        0.5 Check
PLAT300_ALERT_4_G Atom Site Occupancy of H7E        Constrained at        0.5 Check
PLAT300_ALERT_4_G Atom Site Occupancy of H7F        Constrained at        0.5 Check
PLAT302_ALERT_4_G Anion/Solvent/Minor-Residue Disorder (Resd  2  )        50% Note
PLAT302_ALERT_4_G Anion/Solvent/Minor-Residue Disorder (Resd  3  )        67% Note
PLAT367_ALERT_2_G Long?  C(sp?)-C(sp?) Bond  C2       - C3       .       1.51 Ang.
PLAT432_ALERT_2_G Short Inter X...Y Contact  Cl3      ..C6       .       3.04 Ang.
                                                  1+x,y,z  =      1_655 Check
PLAT432_ALERT_2_G Short Inter X...Y Contact  Cl3      ..C6       .       3.04 Ang.
                                                   -x,y,z  =      4_555 Check
PLAT432_ALERT_2_G Short Inter X...Y Contact  Cl4      ..C6       .       3.14 Ang.
                                          1/2-x,1-y,1/2+z  =     10_565 Check
PLAT432_ALERT_2_G Short Inter X...Y Contact  Cl4      ..C6       .       3.14 Ang.
                                            1/2-x,y,1/2+z  =     12_555 Check
PLAT789_ALERT_4_G Atoms with Negative _atom_site_disorder_group  #         25 Check
PLAT860_ALERT_3_G Number of Least-Squares Restraints ............        113 Note
```

```
  0 ALERT level A = Most likely a serious problem - resolve or explain
  1 ALERT level B = A potentially serious problem, consider carefully
 13 ALERT level C = Check. Ensure it is not caused by an omission or oversight
 43 ALERT level G = General information/check it is not something unexpected

  4 ALERT type 1 CIF construction/syntax error, inconsistent or missing data
 16 ALERT type 2 Indicator that the structure model may be wrong or deficient
  2 ALERT type 3 Indicator that the structure quality may be low
 32 ALERT type 4 Improvement, methodology, query or suggestion
  3 ALERT type 5 Informative message, check
```

It is advisable to attempt to resolve as many as possible of the alerts in all categories. Often the minor alerts point to easily fixed oversights, errors and omissions in your CIF or refinement strategy, so attention to these fine details can be worthwhile. In order to resolve some of the more serious problems it may be necessary to carry out additional measurements or structure refinements. However, the purpose of your study may justify the reported deviations and the more serious of these should normally be commented upon in the discussion or experimental section of a paper or in the "special_details" fields of the CIF. checkCIF was carefully designed to identify outliers and unusual parameters, but every test has its limitations and alerts that are not important in a particular case may appear. Conversely, the absence of alerts does not guarantee there are no aspects of the results needing attention. It is up to the individual to critically assess their own results and, if necessary, seek expert advice.

Publication of your CIF in IUCr journals

A basic structural check has been run on your CIF. These basic checks will be run on all CIFs submitted for publication in IUCr journals (*Acta Crystallographica*, *Journal of Applied Crystallography*, *Journal of Synchrotron Radiation*); however, if you intend to submit to *Acta Crystallographica Section C* or *E* or *IUCrData*, you should make sure that full publication checks are run on the final version of your CIF prior to submission.

Publication of your CIF in other journals

Please refer to the *Notes for Authors* of the relevant journal for any special instructions relating to CIF submission.

PLATON version of 28/11/2022; check.def file version of 28/11/2022

Datablock w03300K - ellipsoid plot

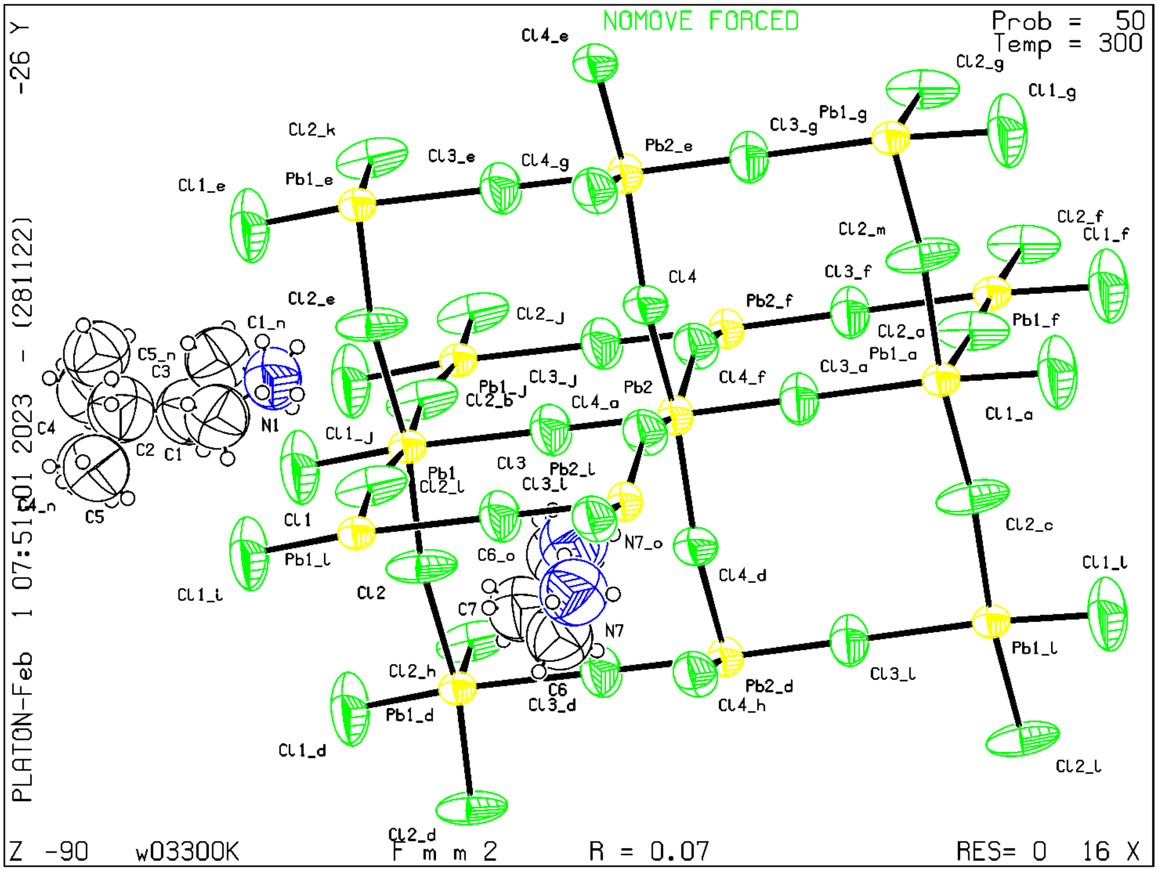

Supplement: Supplementary file 2 — Supporting cif files [file ADVS-10-2301064-s001.zip › w03300K checkcif.pdf]
