# Supplementary material for: Improper High‐T c Perovskite Ferroelectric with Dielectric Bistability Enables Broadband Ultraviolet‐to‐Infrared Photopyroelectric Effects
Source: Adv Sci (Weinh). 2023 Apr 23;10(19):2301064. doi: 10.1002/advs.202301064 (PMC10323668; doi:10.1002/advs.202301064)

checkCIF/PLATON report

You have not supplied any structure factors. As a result the full set of tests cannot be run.

THIS REPORT IS FOR GUIDANCE ONLY. IF USED AS PART OF A REVIEW PROCEDURE FOR PUBLICATION, IT SHOULD NOT REPLACE THE EXPERTISE OF AN EXPERIENCED CRYSTALLOGRAPHIC REFEREE.

No syntax errors found. CIF dictionary Interpreting this report

Datablock: w03400K

Bond precision: Pb-Cl = 0.0019 A Wavelength=0.71073

Cell:	a=5.7493(3)	b=5.7493(3)	c=56.681(5)
	alpha=90	beta=90	gamma=90
Temperature:	400 K		

	Calculated	Reported
Volume	1873.6(3)	1873.5(3)
Space group	I 4/m m m	I 4/m m m
Hall group	-I 4 2	-I 4 2
Moiety formula	C14 H44 Cl10 N4 Pb3	2(Cl5 Pb1.5), 2(C5 H14 N), C4 H16 N2
Sum formula	C14 H44 Cl10 N4 Pb3	C14 H44 Cl10 N4 Pb3
Mr	1244.63	1244.60
Dx,g cm-3	2.206	2.206
Z	2	2
Mu (mm-1)	14.170	14.171
F000	1144.0	1144.0
F000'	1128.19	
h,k,lmax	7,7,73	7,7,73
Nref	749	743
Tmin,Tmax		
Tmin'		

Correction method= Not given

Data completeness= 0.992 Theta(max)= 27.506

R(reflections)= 0.0367(548) wR2(reflections)= 0.1080(743)

S = 1.060 Npar= 90

The following ALERTS were generated. Each ALERT has the format
test-name_ALERT_alert-type_alert-level.
Click on the hyperlinks for more details of the test.

Alert level B

```
PLAT241_ALERT_2_B High   'MainMol' Ueq as Compared to Neighbors of        Cl2 Check
PLAT242_ALERT_2_B Low    'MainMol' Ueq as Compared to Neighbors of        Pb1 Check
PLAT242_ALERT_2_B Low    'MainMol' Ueq as Compared to Neighbors of        Pb2 Check
```

Alert level C

```
PLAT042_ALERT_1_C Calc. and Reported MoietyFormula Strings  Differ     Please Check
PLAT052_ALERT_1_C Info on Absorption Correction Method   Not Given     Please Do !
PLAT053_ALERT_1_C Minimum Crystal Dimension Missing (or Error) ...     Please Check
PLAT054_ALERT_1_C Medium  Crystal Dimension Missing (or Error) ...     Please Check
PLAT055_ALERT_1_C Maximum Crystal Dimension Missing (or Error) ...     Please Check
PLAT088_ALERT_3_C Poor Data / Parameter Ratio ...................        8.32 Note
PLAT260_ALERT_2_C Large Average Ueq of Residue Including       Pb1      0.148 Check
```

Alert level G

```
PLAT002_ALERT_2_G Number of Distance or Angle Restraints on AtSite          9 Note
PLAT003_ALERT_2_G Number of Uiso or Uij Restrained non-H Atoms ...          9 Report
PLAT005_ALERT_5_G No Embedded Refinement Details Found  in the CIF     Please Do !
PLAT007_ALERT_5_G Number of Unrefined Donor-H Atoms .............          6 Report
PLAT232_ALERT_2_G Hirshfeld Test Diff (M-X)  Pb1      --Cl2      .        6.0 s.u.
PLAT300_ALERT_4_G Atom Site Occupancy of N0AA      Constrained at      0.125 Check
PLAT300_ALERT_4_G Atom Site Occupancy of N2        Constrained at      0.125 Check
PLAT300_ALERT_4_G Atom Site Occupancy of C1        Constrained at      0.125 Check
PLAT300_ALERT_4_G Atom Site Occupancy of C0AA      Constrained at      0.125 Check
PLAT300_ALERT_4_G Atom Site Occupancy of C3        Constrained at      0.125 Check
PLAT300_ALERT_4_G Atom Site Occupancy of C4        Constrained at      0.125 Check
PLAT300_ALERT_4_G Atom Site Occupancy of C5        Constrained at      0.125 Check
PLAT300_ALERT_4_G Atom Site Occupancy of C6        Constrained at      0.125 Check
PLAT300_ALERT_4_G Atom Site Occupancy of C7        Constrained at      0.125 Check
PLAT300_ALERT_4_G Atom Site Occupancy of H0AD      Constrained at      0.125 Check
PLAT300_ALERT_4_G Atom Site Occupancy of H1A       Constrained at      0.125 Check
PLAT300_ALERT_4_G Atom Site Occupancy of H1B       Constrained at      0.125 Check
PLAT300_ALERT_4_G Atom Site Occupancy of H1C       Constrained at      0.125 Check
PLAT300_ALERT_4_G Atom Site Occupancy of H0AE      Constrained at      0.125 Check
PLAT300_ALERT_4_G Atom Site Occupancy of H2A       Constrained at      0.125 Check
PLAT300_ALERT_4_G Atom Site Occupancy of H2B       Constrained at      0.125 Check
PLAT300_ALERT_4_G Atom Site Occupancy of H2C       Constrained at      0.125 Check
PLAT300_ALERT_4_G Atom Site Occupancy of H0AA      Constrained at      0.125 Check
PLAT300_ALERT_4_G Atom Site Occupancy of H3A       Constrained at      0.125 Check
PLAT300_ALERT_4_G Atom Site Occupancy of H3B       Constrained at      0.125 Check
PLAT300_ALERT_4_G Atom Site Occupancy of H0AB      Constrained at      0.125 Check
PLAT300_ALERT_4_G Atom Site Occupancy of H4A       Constrained at      0.125 Check
PLAT300_ALERT_4_G Atom Site Occupancy of H4B       Constrained at      0.125 Check
PLAT300_ALERT_4_G Atom Site Occupancy of H5        Constrained at      0.125 Check
PLAT300_ALERT_4_G Atom Site Occupancy of H0AC      Constrained at      0.125 Check
PLAT300_ALERT_4_G Atom Site Occupancy of H6A       Constrained at      0.125 Check
PLAT300_ALERT_4_G Atom Site Occupancy of H6B       Constrained at      0.125 Check
PLAT300_ALERT_4_G Atom Site Occupancy of H6C       Constrained at      0.125 Check
```

```
PLAT300_ALERT_4_G Atom Site Occupancy of H7A        Constrained at      0.125 Check
PLAT300_ALERT_4_G Atom Site Occupancy of H7B        Constrained at      0.125 Check
PLAT300_ALERT_4_G Atom Site Occupancy of H7C        Constrained at      0.125 Check
PLAT301_ALERT_3_G Main Residue  Disorder .............(Resd  1  )         16% Note
PLAT304_ALERT_4_G Non-Integer Number of Atoms in ..... (Resd  1  )     201.56 Check
PLAT720_ALERT_4_G Number of Unusual/Non-Standard Labels .........           7 Note
PLAT764_ALERT_4_G Overcomplete CIF Bond List Detected (Rep/Expd) .       1.58 Ratio
PLAT789_ALERT_4_G Atoms with Negative _atom_site_disorder_group  #         31 Check
PLAT811_ALERT_5_G No ADDSYM Analysis: Too Many Excluded Atoms ....          ! Info
PLAT860_ALERT_3_G Number of Least-Squares Restraints ............          91 Note
```

```
   0 ALERT level A = Most likely a serious problem - resolve or explain
   3 ALERT level B = A potentially serious problem, consider carefully
   7 ALERT level C = Check. Ensure it is not caused by an omission or oversight
  43 ALERT level G = General information/check it is not something unexpected

   5 ALERT type 1 CIF construction/syntax error, inconsistent or missing data
   7 ALERT type 2 Indicator that the structure model may be wrong or deficient
   3 ALERT type 3 Indicator that the structure quality may be low
  35 ALERT type 4 Improvement, methodology, query or suggestion
   3 ALERT type 5 Informative message, check
```

It is advisable to attempt to resolve as many as possible of the alerts in all categories. Often the minor alerts point to easily fixed oversights, errors and omissions in your CIF or refinement strategy, so attention to these fine details can be worthwhile. In order to resolve some of the more serious problems it may be necessary to carry out additional measurements or structure refinements. However, the purpose of your study may justify the reported deviations and the more serious of these should normally be commented upon in the discussion or experimental section of a paper or in the "special_details" fields of the CIF. checkCIF was carefully designed to identify outliers and unusual parameters, but every test has its limitations and alerts that are not important in a particular case may appear. Conversely, the absence of alerts does not guarantee there are no aspects of the results needing attention. It is up to the individual to critically assess their own results and, if necessary, seek expert advice.

Publication of your CIF in IUCr journals

A basic structural check has been run on your CIF. These basic checks will be run on all CIFs submitted for publication in IUCr journals (*Acta Crystallographica*, *Journal of Applied Crystallography*, *Journal of Synchrotron Radiation*); however, if you intend to submit to *Acta Crystallographica Section C* or *E* or *IUCrData*, you should make sure that full publication checks are run on the final version of your CIF prior to submission.

Publication of your CIF in other journals

Please refer to the *Notes for Authors* of the relevant journal for any special instructions relating to CIF submission.

PLATON version of 28/11/2022; check.def file version of 28/11/2022

Datablock w03400K - ellipsoid plot

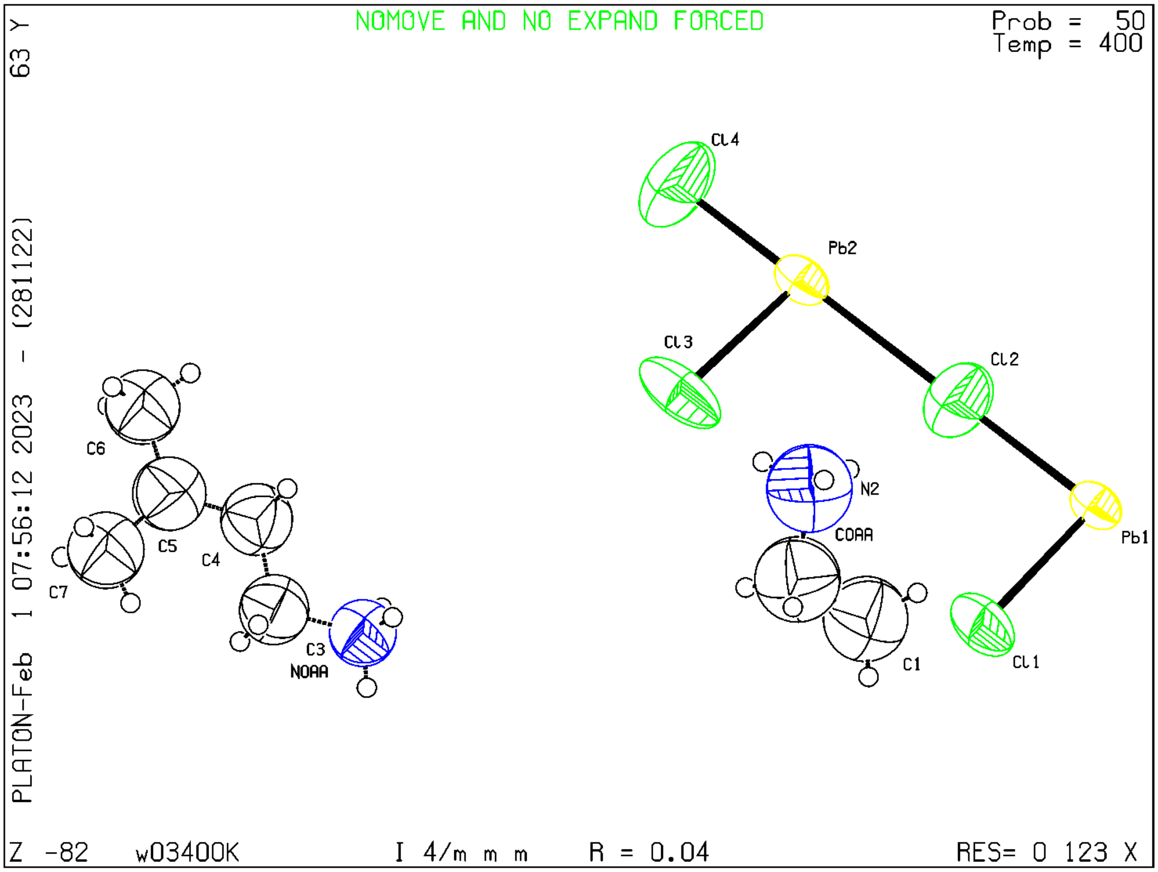

Supplement: Supplementary file 2 — Supporting cif files [file ADVS-10-2301064-s001.zip › w03400K checkcif.pdf]
